# Can Enjoyment and Physical Self-Perception Mediate the Relationship between BMI and Levels of Physical Activity? Preliminary Results from the Regional Observatory of Motor Development in Italy

**DOI:** 10.3390/ijerph191912567

**Published:** 2022-10-01

**Authors:** Domenico Monacis, Athos Trecroci, Pietro Luigi Invernizzi, Dario Colella

**Affiliations:** 1 Department of Humanities, Letters, Cultural Heritage, Education Sciences, University of Foggia, 71121 Foggia, Italy; 2 Department of Biomedical Sciences for Health, Università degli Studi di Milano, 20110 Milano, Italy; 3 Department of Biological and Environmental Sciences and Technologies, University of Salento, 73100 Lecce, Italy

**Keywords:** BMI, enjoyment, physical activity, self-perception, teaching styles

## Abstract

Physical education during adolescence, supported by evidenced-based methodologies, offers many different opportunities to practice structured physical activity and promote the development of motor skills, motor coordination, and conditioning. The present study aimed to assess differences in the levels of physical activity, enjoyment, and self-perception in a sample (*n* = 1029, M = 505, F = 524) of 11–12-year-old secondary schoolchildren according to gender and BMI and determine (a) the mediation effects of physical self-perception in the association between BMI and physical activity and (b) the role of enjoyment in mediating the relation between physical self-perception and physical activity. As part of the Regional Observatory of Motor Development Project (Apulia, Southern Italy), the assessment involved three questionnaires for physical activity levels (PAL), physical self-perception (PSP_C), and enjoyment (PACES). The results showed significant differences in PSP between normal-weight, overweight, and obese children (especially in girls), while there were no significant differences in enjoyment. Physical self-perception partially mediates the relationship between BMI and PAL (R^2^ = 7.94% for males, 95% C.I.: −0.013, −0.004; R^2^ = 14.70% for females, 95% C.I.: −0.25, −0.009), and the enjoyment partially mediates the relationship between physical self-perception and PAL (R^2^ = 6.83% for males, 95% C.I. = 0.003, 0.012; R^2^ = 13.45% for females, 95% C.I. = 0.002, 0.014). However, only a small percentage of variance was explained, precluding the extension and generalization of the results obtained.

## 1. Introduction

The promotion of physical activity represents a global and public health problem for children and adolescents. According to the latest guidelines of the World Health Organization (WHO) [1], structured and unstructured daily physical activity can promote children’s health status through cognitive, motor, social, and personal development. At all ages, sedentary habits and physical inactivity represent important risk factors for health as the main causes of non-communicable diseases [1].

Guthold et al. (2020), in a recent survey study based on a sample of 1.6 million subjects from 146 countries, noted that (a) over 80% of boys between 11 and 17 years do not follow WHO recommendations to perform at least one hour of physical activity per day, and (b) girls are more inactive than boys (85%, compared to 78%) [2]. The authors analyzed the data collected through schools, and the assessment included all kinds of physical activity: active games, recreational and sports activities, household chores, walking, cycling, or other types of active transport, in addition to hours of physical education.

Physical inactivity is one of the most important public health problems of the 21st century, and consequently, different institutional interventions are also needed to promote active lifestyles through different and numerous opportunities for motor activities in different contexts [3].

Physical and sports education offer a significant and distinctive contribution to motor, cognitive, emotional, and social development, as well as to the development of motor skills during childhood, which are precursors for healthy lifestyles and future adherence to physical activity and sport [4]. Although recent evidence suggests the need to develop integrated and multi-component interventions in educational contexts (school, health, and start-up sports), encouraging the practice of physical activity and its beneficial effects on academic achievement and psycho-social development [5], children and adolescents spend most of the day in school, and this may mean less time for physical education and active play [6]. An increase in daily physical activity and a decrease in sedentary behavior require concrete educational actions with positive effects on health promotion during the developmental stage [1,7].

Physical education during adolescence, supported by evidenced-based methodologies, offers many different opportunities to practice structured physical activity and promote the development of motor skills, motor coordination, and conditioning [8]. The significant contribution of motor experiences to the development and interaction of the organic, affective, social, and cognitive areas is crucial to promote the health education of young people through physically active lifestyles [4,9].

“*Physical Literacy*” is the term used to define the entire process of motor and bodily experiences lived by children, aimed at integrating motor skill learning, knowledge, behaviors, and intrinsic motivation, promoting the adoption of active lifestyles during their lifetime [10]. Conversely, *Physical Illiteracy* defines a lack of motivation and self-perception and a reduced repertoire of motor skills, part of the *Pediatric Inactivity Triad*, also including pediatric dynapenia and exercise deficit disorder [11]. The interaction between these variables leads to a vicious circular process, in which lower levels of moderate-to-vigorous physical activity (MVPA) imply less participation in physical activities (even free/unstructured ones) and low levels of physical fitness and efficiency. This condition undermines the achievement of the positive state of mind and enjoyment associated with body movement [11].

Self-perception and enjoyment are key factors in promoting motor learning and motor development, providing better adherence to physical activity [12,13]. All motor and bodily experiences performed by children improve self-perception, that is, the confidence regarding the ability to successfully master a skill [14]. This is also linked to one’s own body perception [15] and the ability to mobilize cognitive, motor, and social resources to perform motor skills in different contexts and everyday activities [13]. According to Khodaverdi et al. (2015) and Dapp et al. (2019), the teaching−learning process and the quality of the motor experience can enhance physical self-perception, mediating the relation between cognitive, motor, emotional, and social functions [16,17]. Pleasant motor experiences lived *with* and *through* body movement are positively associated with the development of enjoyment and self-perception [8]. These factors are closely related, as pleasant and fun motor experiences determine a greater awareness of the practice of motor activities in different contexts and at different ages [18]. Studies have reported lower levels of physical self-perception and enjoyment in overweight and obese children than in normal-weight ones [19,20]. Of note, Vaquero-Solìs et al. (2021) suggested the important role of self-perception and happiness in mediating the relation between physical activity and quality of life, regardless of BMI [21]. The study conducted by Fernández-Bustos et al. (2019) highlights the mediating role of BMI between physical self-perception and the levels of physical activity [22]. In a recent systematic review, Trecroci et al. (2021) highlighted the inverse relationship between perceived motor competence/actual motor competence (as the perception of what a child can do and what a child does, respectively) and BMI and levels of physical activity [23]. Additionally, another systematic review and meta-analysis showed the significant effects of physical activity in enhancing enjoyment, perceived autonomy, and intrinsic motivation [24]. Moreover, enjoyment during physical activity is a powerful moderator of the relationship between physical activity and psychological distress in overweight/obese adolescents [25]. Besides weight status factors, the importance of psychological factors, such as enjoyment and self-perception, in driving differences in adherence to physical activity and healthy lifestyles requires an accurate analysis of the environment in which children move and play and, more generally, of the entire teaching−learning process. The Regional Observatory of Motor Development Project in Apulia Region (Southern Italy) aims to systematically assess physical fitness and its correlates with physical activity and to monitor children’s and adolescents’ health status: the data collected can be used in scientific research to develop appropriate physical education practices and national institutional guidelines. Moreover, it would offer the opportunity to periodically acquire quanti-qualitative data on the evolution of physical fitness and health-related components in different geographical areas to assess the effectiveness of scholastic, institutional, and sport interventions aimed at promoting physical activity.

Therefore, the present study, after assessing differences in the levels of physical activity, enjoyment, and self-perception in a sample of 11−12-year-old secondary school children according to gender and BMI and a significant correlation between anthropometric characteristics and psychological correlates, aimed to determine (as a primary outcome) whether (a) physical self-perception mediates the relation between BMI and physical activity and whether (b) enjoyment is a mediating variable between physical self-perception and levels of physical activity. A mediation analysis between correlated variables was performed according to male and female gender and for the total sample.

The hypotheses of the study are that (1) better self-perception represents an important mediating factor between BMI and the levels of physical activity (children who have better perceptions of their body image and competence are more active) and that (2) greater enjoyment during the practice of motor activity can contribute to increasing PAL and adds further benefits to the children’s health status.

## 2. Materials and Methods

### 2.1. Sample

The following study was coordinated by the University of Foggia (Italy), Degree in Sciences and Techniques of Preventive and Adapted Motor Activities, Laboratory of Didactic of Motor Activities, sponsored by the Apulia Region. The sample (*n* = 1029) was recruited by schools that joined the Regional Observatory of Motor Development project, and this preliminary study involved schoolchildren from the Apulian province only (Foggia, which is the project leader). Sample recruitment was performed via a simple randomization procedure.

The total sample was divided according to gender (male and female) and group (normal weight, overweight, and obese). Table 1 summarizes the sample’s anthropometric characteristics.

### 2.2. Procedure and Measures

The assessment occurred during curricular physical education lessons and was conducted by a team of experts in Motor and Sports Sciences and in Preventive and Adapted Motor Activities, PhD students, and physical education teachers involved in the Regional Observatory of Motor Development Project. Training meetings were organized for all university staff and teachers to standardize assessment methods in the Laboratory of Didactic of Motor Activities in order to verify the validity, objectivity, and reliability of data. During the meetings, all staff involved participated in training groups, in which, in addition to the description of the anthropometric assessment and physical fitness tests, they were asked to evaluate each other, highlighting any critical issues in the assessment process. It was also shown how to record data in a spreadsheet for data management.

Informed consent was obtained for data collection, as required by the research procedure at the University of Foggia [26].

After anthropometric data collection (age, weight, height, and BMI), Cole’s Scale [27] was used to classify children as normal weight (Nw), overweight (Ow), or obese (Ob).

Cole’s Scale is an assessment tool that defines children’s normal weight, overweight, and obese status using cutoff points based on BMI centile curves and is adjusted by age and sex [27].

Then, the following questionnaires were administered to assess the levels of physical activity, enjoyment, and physical self-perception, respectively:

- The Physical Activity Questionnaire for Older Children (PAQ-C) [28,29,30]: It is a questionnaire aimed at assessing physical activity levels in the last 7 days, including sports activities, recreational activities, dancing, climbing, and cycling but also unstructured recreational activities, in children aged 8−14 years. Low scores (from 1 to 2.33) correspond to poor PAL, average scores (from 2.34 to 3.66) indicate moderate PAL, and higher scores (from 3.67 to 5.00) imply higher PAL.
- The Physical Activity Enjoyment Scale (PACES) [31]: It is a 16-item questionnaire, using a 5-point Likert scale (1 = in complete disagreement; 2 = in disagreement; 3 = uncertain; 4 = in agreement; 5 = in full agreement) in children and adolescents aged 11−19 years. It is composed of two sub-scales: PACES_P (positive feelings) and PACES_N (negative feelings). Only the positive scale was used in this study.
- The Physical Self-Efficacy Scale for Children [32]: It is a 6-item questionnaire based on a 4-point Likert scale (max 24 points) that investigates children’s perception of strength, speed, and coordination (for children aged 10−20 years). High scores are indices of higher levels of self-perception, while lower scores imply lower ones.

### 2.3. Statistical Analysis

**Figure 1:**
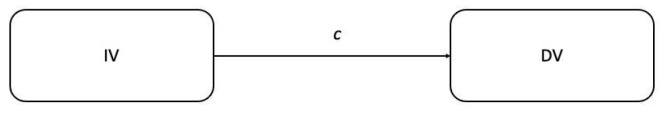
Model without the mediation variable; *c* = total effect.

**Figure 2:**
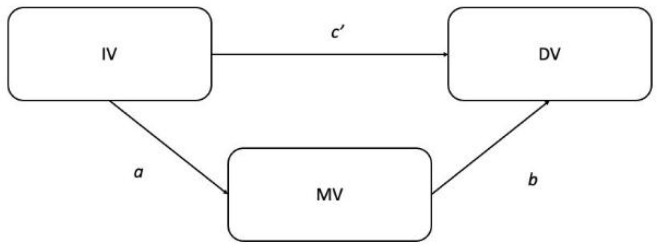
Model with the addition of mediation variable; *c*’ = direct effect; *a* = effect of IV on MV; *b* = effect of MV on DV.

In addition to descriptive statistics (mean ± standard deviation), the Shapiro-Wilk test was performed to verify normal data distributions. An analysis of variance (ANOVA) was carried out to highlight differences between variables, and Tuckey’s Post Hoc test was performed to describe differences between normal weight, overweight, and obese according to gender (male and female) and for the total sample. Pearson’s correlation coefficient (*r*) was calculated to analyze significant relations between variables, interpreted as follows: *r* < 0.30 = weak relationship; 0.30 < *r* < 0.70 = moderate relationship; *r* > 0.070 = strong relationship). Then, the mediation model was applied to understand the relationship between BMI, physical activity levels, and related factors. The analysis of mediation factors involves the interaction of 3 variables, the independent variable (IV), dependent variable (DV), and mediation variable (MV), in order to understand the effect of IV on DV, partially or totally caused by MV. Following the method described by Baron and Kenny (1986), the following operations were required to estimate the mediation effects: (1) simple linear regression of IV on DV; (2) simple linear regression of IV on MV; and (3) multiple linear regression of MV and IV over DV [33]. To be considered effective, the study of mediation factors must meet the following assumptions: (a) the regression coefficients of steps 1 and 2 must be significant (otherwise, no further analysis is possible); (b) the mediator must be a significant predictor of DV in step 3, and (c) the regression coefficient of IV over DV in step 3 must be lower than that of step 1. The method subsequently introduced by Preacher and Hayes (2004) allows these regression equations to be estimated and the “indirect effect” to be considered as a new criterion for establishing mediation [34]. Specifically, it is possible to calculate:

- The total effect (*c*): effect of IV on DV (Figure 1);
- The direct effect (*c’*): effect of IV on DV controlled per MV;
- The indirect effect (*a•b*) = the product of the effects of IV on MV (*a*) and MV on DV (*b*; Figure 2).

If the indirect effect is significant, the mediation is defined as partial; if it is not significant, it is defined as total. The Bootstrapping method of Preacher and Hayes [34,35] was used to evaluate the significance of mediations. All of the results with a *p*-value < 0.05 were considered significant. Data analysis was performed with SPSS version 25.0 (IBM Corp., Armonk, NY, USA) for Windows.

## 3. Results

**Figure 3:**
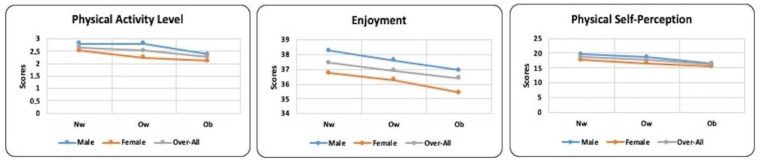
Levels of physical activity, enjoyment, and physical self-perception in males, females, and overall sample according to BMI cutoff.

No missing cases were reported in this study. All of the children recruited carried out the anthropometric assessment and completed the questionnaires provided. The results of ANOVA (Table 2) showed no statistically significant differences in physical activity levels between normal-weight and overweight groups in the male sample, while a significant difference was observed between the Nw and Ob groups and between the Ow and Ob groups. For girls, the normal-weight group was more active than both the overweight and obese groups, while no significant differences were found between the overweight and obese groups. However, the results for the total sample showed a progressive decline in the levels of physical activity, with significant differences between all groups. Figure 3 (referring to physical activity levels) shows that as the BMI increases, physical activity levels decrease, especially among obese children.

Although enjoyment values decreased from normal weight to overweight and obese, there were no statistically significant differences in any group (i.e., male, female, and total sample) (Figure 3). The results of self-perception showed higher values in normal weight compared to overweight and obese in all groups (i.e., male, female, and total sample). There were no significant differences between overweight and obese girls. Figure 3 shows the negative trend of self-perception in relation to the BMI cutoff.

Table 3 shows the correlation coefficients between the considered variables (BMI, PAL, enjoyment, and self-perception). The results showed an inverse weak relationship between BMI and physical activity levels in all groups (Male: *r* = −0.199, *p* < 0.01; Female: *r* = −0.244, *p* < 0.01; Total Sample: *r* = −0.203, *p* < 0.01), as well as for self-perception (Male: *r* = −0.264, *p* < 0.01; Female: *r*= −0.213, *p* < 0.01; Total Sample: *r* = −0.226, *p* < 0.01). There was no significant correlation between BMI and enjoyment in any group. Correlation analysis also showed a statistically significant weak relationship between PAL and enjoyment (Male: *r* = 0.169, *p* < 0.01; Female: *r* = 0.232, *p* < 0.01; Total Sample: *r* = 0.220, *p* < 0.01), weak to moderate relation between PAL and self-perception (Male: *r* = 0.245, *p* < 0.01; Female: *r* = 0.346, *p* < 0.01; Total Sample: *r* = 0.329, *p* < 0.01), and moderate one between enjoyment and self-perception (Male: *r* = 0.337, *p* < 0.01; Female: *r* = 0.384, *p* < 0.01; Total Sample: *r* = 0.375, *p* < 0.01).

**Figure 4:**
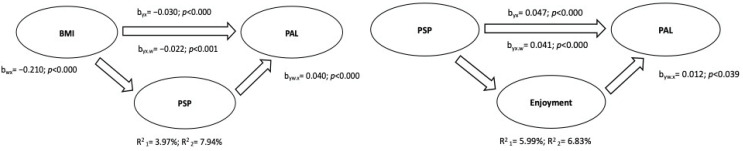
Mediation Analysis of Male Sample.

**Figure 5:**
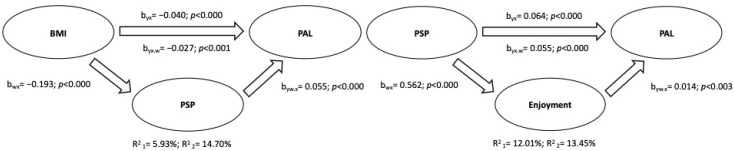
Mediation Analysis of Female Sample.

**Figure 6:**
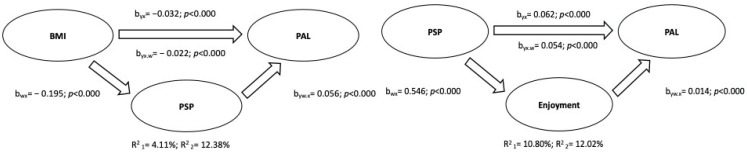
Mediation Analysis of Total Sample.

The first mediation model was structured by placing BMI as IV, physical activity levels as DV, and self-perception as MV (based on the correlation coefficients shown in Table 3). The mediation analysis between correlated variables was performed according to gender (male and female) and the total sample. The results obtained from the mediation analysis are shown in schematic form in Figure 4, Figure 5 and Figure 6.

The results can be summarized as follows: (a) in all groups (male, female, and total sample), BMI was inversely related to the physical activity level, explaining 3.97% (95% C.I.: −0.04, −0.02), 5.93% (95% C.I.: −0.05, −0.02), and 4.11% (95% C.I.: −0.04, −0.02) of the variance in males, females, and the total sample, respectively; (b) physical self-perception partially mediated the relation between BMI and the physical activity level, and the variance explained by the addition of the mediation variable was R^2^ = 7.94% for males (95% C.I.: −0.013, −0.004), R^2^ = 14.70% for females (95% C.I.: −0.25, −0.009), and R^2^ = 12.38% (95% C.I.: −0.015, −0.007) for the total sample.

In addition, the correlations from Table 3 allowed for a structured second mediation model, placing self-perception as IV, the levels of physical activity as DV, and enjoyment as MV. The results highlighted the positive effects of self-perception on the physical activity level (R^2^ = 5.99% for males, 95% C.I. = 0.031, 0.063; R^2^ = 12.01% for females, 95% C.I. = 0.048, 0.078; R^2^ = 10.80% for the total sample, 95% C.I. = 0.051, 0.070), partially mediated by enjoyment, resulting in an increase in total variance explained (R^2^ = 6.83% for males, 95% C.I. = 0.003, 0.012; R^2^ = 13.45% for females, 95% C.I. = 0.002, 0.014; R^2^ = 12.02% for the total sample, 95% C.I. = 0.003, 0.012).

## 4. Discussion

The present study showed significant differences in PAL and PSP between the normal-weight, overweight, and obese samples, especially in girls, while there were no significant differences in enjoyment. This study partially confirms previous results obtained in the Apulian Regional Observatory Project [19,20]. Similar results were also obtained in the study by Baños et al. (2021), highlighting that enjoyment during physical education is closely related to the quantity and quality of time spent in physical activity during leisure time [36]. However, the most interesting results relate to the analysis of the proposed mediation models.

According to the first mediation model, PSP partially mediates the relationship between BMI and PAL (more evident in girls than in boys), and enjoyment partially mediates the relationship between physical self-perception and PAL. The results of the second proposed mediation model, however, although significant, help to explain a small percentage of additional variability explained, precluding the extension and generalization of the results obtained. Other studies have analyzed mediation effects to understand which variables may be involved in determining an increase in the levels of physical activity. A recent systematic review of the literature conducted by Foley Davelaar (2021) clarified the role of BMI and the perception of body image in perceived physical competence: BMI becomes a determining factor in the perception of body image for 6−11 years, while during adolescence, body image becomes a determining factor in ensuring greater adherence to physical activity [37].

Obesity and physical illiteracy, associated with low levels of physical activity, represent a real barrier for children and adolescents, involving the affective, behavioral, physical, and cognitive domains: structured interventions of evidence-based physical education programs could balance the potential negative effects of motor illiteracy and obesity throughout childhood and adolescence [23]. The study by Dishman et al. (2019) highlighted a decline in self-perception that would drive young people to be less active, reducing daily physical activity (overestimating barriers to physical activity), enjoyment, physical fitness, or social goals [38]. De Souza et al. (2021) analyzed the relationship between physical activity, enjoyment, and self-perception and school achievement in a sample of 442 secondary school students aged 11−17 years: better school achievement was positively correlated and associated with higher levels of physical activity and enjoyable physical activity [39]. Kokkonen et al. (2020) analyzed the relationship between the motivational climate in PE, social skills in PE, and out-of-school physical activity in a sample of 363 students aged 9−12 years [40]. The model showed that both social skills and the motivational climate are important tools to increase the motivation to practice physical activity and MVPA, revealing an interesting key to understanding what behavior the teacher should adopt to promote social skills, collaboration, and a positive motivational climate. Another study analyzed the relationship between physical self-perception, both intrinsic and extrinsic motivation, and motor performance in a sample of 1082 children aged 7−8 years [41]. The main findings suggested the key roles assumed by intrinsic motivation and self-perception in improving motor performance and the levels of physical activity during the developmental stage: interventions aimed at improving only quantitative motor factors (jump height, resistance, time spent in physical activity, etc.) may produce fewer or no effects without supporting children’s physical self-perception and intrinsic motivation.

Similarly, according to Meyer, Grob, and Gerber (2021), intrinsic motivation plays a key role in reducing stress and increasing the quality of life through physical activity in adolescents [42]. Moreover, enjoyment during physical education seems to have a mediating effect between autonomy support and physical education performance [43].

In the field of physical education, the teacher is the main actor able to promote one or the other process: physical literacy or illiteracy can be considered the result of the didactic approach and the mediation between the teacher, the student, the various motor tasks and organizational methods, and the setting in which the motor experiences are realized. Recent studies have shown that many of these benefits are not necessarily the result of participation in activities, but the effects could be mediated by the nature of interactions between pupils, teachers, parents, and school employees in both family and socio-cultural contexts [4,44,45]. Physical activity offers significant benefits to school results, cognitive functions, and classroom behavior, affecting the person globally [46,47].

The study of mediation factors for physical education teaching is an important current theme. The term mediation in teaching is commonly used to indicate the incidence of a variable that interposes between two or more variables to facilitate the relationship. In the field of social sciences and education, it takes on meaning in relation to learning processes [48]. Learning is not the result of direct exposure to certain situations (in a simplistic relationship of cause and effect), but it takes place through the interaction of one or more variables. The teacher guides and intentionally defines what behavior to adopt, what communication and teaching styles to use, through which organizational modality to propose the motor tasks, etc. The model of educational mediation or of the learning experiences mediated by the teacher, therefore, is focused on the study, analysis, and acquisition of awareness of the teacher’s behavior to (a) establish an effective educational relationship with pupils, (b) provide optimal conditions to facilitate the achievement of certain learning aims, and (c) promote the social inclusion and participation of all pupils.

Motor activities in the school setting, during both curricular and extracurricular hours, increase the quanti-qualitative opportunities for motor skill learning and development, generating a circular process that is a necessary precursor to proper lifestyles and sports participation. In the school setting, the interdisciplinarity of learning contributes to promoting wellbeing and preventing non-communicable diseases through inter-institutional and multi-component interventions that require the monitoring of processes and results. The systematic assessment of physical activity and physical fitness allows the acquisition and analysis of the transversal and longitudinal data necessary to customize the educational process [8].

The assessment of curricular and extracurricular intervention outcomes is unavoidable when studying the effects of the educational process and analyzing relationships with related factors in different periods of the school year, sharing and disseminating results to different educational agencies.

## 5. Methodological and Didactic Implications

The findings suggest that school-based or extracurricular interventions targeted at improving children’s physical activity should not be focused on physical activity levels only. Indeed, enjoyment, physical self-perception, body image, and intrinsic and extrinsic motivation are factors that contribute to determining both the process of learning and motor development of the child, but also the way in which the child participates in physical activity, socializes, and interacts with their teacher and peers.

A key role of great responsibility is attributed to teachers, who are able to provide significant motor experiences that encourage the continuation of motor practice in out-of-school contexts, as well. This would promote an increase in a child’s levels of daily physical activity and motor development. The organization of the educational setting, in fact, is closely associated with the way in which the teacher intentionally proposes a certain motor task, defining what to do and how to do it through the variation in teaching styles [49] by making a series of statements and questions that allow the teacher (a) to intentionally promote specific factors (e.g., enjoyment, self-perception, development of specific motor skills and abilities, etc.) and (b) to adapt, leaving more or less decision-making autonomy, the task in relation to the student’s individual motor repertoire and competence.

The study of the mediation factors allows for identifying variables that develop (accelerate/inhibit) a process with important effects on the methodological and didactic level. Once the accelerator factor is known, the teacher, through the variation in teaching styles, can intervene in the didactic setting, orienting the modalities of learning and intentionally promoting specific socio-affective and relational components [49].

The variation in and use of different teaching styles could increase physical fitness levels, motor competence, enjoyment, and time spent in physical activity, promoting healthy lifestyles and physical literacy in primary school children [50].

In practice, the teacher, through the knowledge of which factors accelerate or inhibit some process in a certain learning stage, can manage the relation between motor task complexity, the performer’s ability, and the conditions of practice [49].

From an evidence-based didactic perspective, the teaching process concerns not only the definition of disciplinary areas and core themes (e.g., playing with small tools, body expression and movement, sports, team games, etc.) but also the choice of contents, motor tasks, and organizational methods enriched by the knowledge of the teaching styles and didactic approaches that can best promote motor learning and motor development. The teaching−learning process is necessarily carried out through a wide repertoire of contents and organizational methods, but it develops and proceeds beyond this context, mobilizing different factors that structure motor competence itself, which highlights the teacher’s mediating role [19,20,23,36].

Yap (2021) focused on the relationship between motivation factors and the variability of teaching styles, recommending the implementation of physical education programs and interventions that use different teaching styles [51] to activate different areas of the person (motor, emotional, and cognitive) [52]. However, findings in the field of physical education show that reproduction teaching styles (i.e., practice and command) are predominantly used at the expense of the production styles (i.e., problem-solving- and discovery-guided) [53,54].

This requires careful analysis and reflection, not only on the number of opportunities for children to be physically active but also on the quality of motor experiences lived in school and out of school and on teachers’ training. It is necessary to structure physical education and motor activity programs based on motor task variability, carried out in different organizational ways and presented with appropriate teaching styles.

## 6. Conclusions

This study emphasizes the relationship between BMI and PAL and the importance of PSP and enjoyment in enhancing PAL.

The results of the present study could be useful to develop best practices and provide support for PE teachers in structuring the didactic-educational process: it should not be forgotten that the educational action of the PE teacher is translated into the motor tasks proposed.

Therefore, the results of the study can be interpreted as follows: the knowledge of which factors (enjoyment and self-perception) are more or less developed in different groups (normal weight, overweight, and obese) allows the teacher to reflect on the motor tasks he or she proposes, choosing how to change and vary the motor task in a way that involves all children, motivating students and increasing, as evidenced in this study, the levels of physical activity for health promotion.

In fact, the proposal of motor tasks and the choice of organizational and communicative methods, together with the variation in productive (indirect) and reproductive (direct) teaching styles—centered on the students and the teachers, respectively—assume a fundamental role in the structuring of the teaching−learning process.

Identifying, distinguishing, and analyzing the relationships between the motor skills, motor abilities, executive variants, and tools used within a motor task, contextualized in certain organizational modes and proposed using appropriate teaching styles, are the teacher’s inescapable skills in the teaching of motor activities.

The combination of parameters such as intensity, the difficulty in execution, and the density of the proposed tasks causes different effects, ranging from the pronounced stress of some skills to the learning of more or less complex skills and the overall involvement of the person, in which self-perception, motivation, socialization, and enjoyment take on particular importance.

The teacher’s goal is, therefore, to promote didactic obliquity, that is, to modulate the executive difficulty of a motor task within the stages of the individual’s proximal development within a given context (organizational modalities and teaching styles) so that it is pleasant and amusing while mobilizing the psycho-physical resources necessary to complete and/or correctly perform that given task. Conversely, tasks that are too simple or too difficult do not induce an optimal state of activation, and this involves the progressive removal of the practice of physical activity.

The limits of the present study concern the small variance explained by both mediation models, so the results obtained cannot be generalized. Further research should aim to better understand the role of physical self-perception and enjoyment in determining physical activity levels according to different variables, such as the geographic area (urban or rural city), age (primary or secondary school children, etc.), BMI (normal weight, overweight, and obese), and athleticism (athletes and non-athletes).

## Figures and Tables

**Table 1:** Anthropometric characteristics of the sample.

Gender	Group	N	Age	Weight	Height	BMI
Male	Nw	278	12.07 ± 0.81	43.68 ± 9.06	1.54 ± 0.11	18.05 ± 2.02
	Ow	142	12.01 ± 0.77	57.43 ± 9.01	1.56 ± 0.09	23.18 ± 1.55
	Ob	85	11.87 ± 0.80	72.07 ± 11.16	1.57 ± 0.11	28.97 ± 3.42
Female	Nw	319	11.99 ± 0.84	44.14 ± 8.29	1.54 ± 0.08	18.48 ± 2.31
	Ow	157	12.10 ± 0.82	57.70 ± 7.46	1.55 ± 0.08	23.63 ± 1.47
	Ob	48	11.94 ± 0.86	70.16 ± 11.66	1.55 ± 0.09	29.00 ± 3.05
Overall	Nw	597	12.03 ± 0.83	43.92 ± 8.65	1.54 ± 0.10	18.28 ± 2.19
	Ow	299	12.05 ± 0.80	57.58 ± 8.22	1.56 ± 0.10	23.42 ± 1.53
	Ob	133	11.89 ± 0.82	71.39 ± 11.33	1.56 ± 0.10	28.98 ± 3.30

N = number of subjects; BMI = body mass index; Nw = normal weight; Ow = overweight; Ob = obese.

**Table 2:** Measures of Physical Activity Level (PAL), Enjoyment, and Physical Self-Perception (PSP).

		Nw		Ow		Ob		Post Hoc Test		
Male		M	SD	M	SD	M	SD			
	PAL	2.81	0.69	2.81	0.73	2.38	0.66	Nw = Ow	Nw > Ob **	Ow > Ob **
	Enjoyment	38.25	4.99	37.61	5.67	36.92	6.98	Nw = Ow	Nw = Ob	Ow = Ob
	PSP	19.60	3.34	18.72	3.48	16.38	4.16	Nw > Ow *	Nw > Ob **	Ow > Ob **
Female										
	PAL	2.52	0.64	2.25	0.60	2.10	0.57	Nw > Ow **	Nw > Ob **	Ow = Ob
	Enjoyment	36.73	6.23	36.28	5.30	35.42	5.24	Nw = Ow	Nw = Ob	Ow = Ob
	PSP	17.74	3.57	16.67	3.12	15.52	4.09	Nw > Ow **	Nw > Ob **	Ow = Ob
Overall										
	PAL	2.66	0.70	2.52	0.72	2.28	0.65	Nw > Ow *	Nw > Ob **	Ow > Ob **
	Enjoyment	37.44	5.73	36.90	5.51	36.38	6.43	Nw = Ow	Nw = Ob	Ow = Ob
	PSP	18.61	3.60	17.64	3.45	16.07	4.15	Nw > Ow **	Nw > Ob **	Ow > Ob **

Nw = normal weight, Ow = overweight, Ob = obese: * = *p* < 0.05; ** = *p* < 0.01.

**Table 3:** Pearson’s correlation coefficients among variables. ** = *p* < 0.01.

Group	Factors									
		PAL			Enjoyment			PSP		
		r	C.I.		r	C.I.		r	C.I.	
Male	BMI	−0.199 **	−1.84	−0.73	−0.073	−0.13	0.01	−0.264 **	−0.43	−0.22
	PAL				0.169 **	0.01	0.03	0.245 **	0.03	0.06
	Enjoyment							0.337 **	0.38	0.63
		PAL			Enjoyment			PSP		
		r	C.I.		r	C.I.		r	C.I.	
Female	BMI	−0.244 **	−2.07	−1.01	−0.036	−0.08	0.03	−0.213 **	−0.33	−0.14
	PAL				0.232 **	0.01	0.03	0.346 **	0.04	0.07
	Enjoyment							0.384 **	0.50	0.76
Over-All		PAL			Enjoyment			PSP		
		r	C.I.		r	C.I.		r	C.I.	
	BMI	−0.203 **	−1.63	−0.88	−0.050	−0.84	0.009	−0.226 **	−0.33	−0.20
	PAL				0.220 **	0.20	0.035	0.329 **	0.05	0.07
	Enjoyment							0.375 **	0.49	0.67

## Data Availability

We guarantee that the shared data complies with the consent of the participants on the use of confidential data.
